# A cut-off of daily sedentary time and all-cause mortality in adults: a meta-regression analysis involving more than 1 million participants

**DOI:** 10.1186/s12916-018-1062-2

**Published:** 2018-05-25

**Authors:** Po-Wen Ku, Andrew Steptoe, Yung Liao, Ming-Chun Hsueh, Li-Jung Chen

**Affiliations:** 1 Graduate Institute of Sports and Health, National Changhua University of Education, Changhua City, Taiwan; 2 Department of Behavioural Science and Health, University College London, London, UK; 3 Department of Health Promotion and Health Education, National Taiwan Normal University, Taipei, Taiwan; 4 Department of Physical Education, National Taiwan Normal University, NO.162, He-ping East Road, Section 1, Taipei, 106 Taiwan; 5 Department of Exercise Health Science, National Taiwan University of Sport, No. 16, Section 1, Shuang-Shih Rd., Taichung, 404 Taiwan

**Keywords:** Sedentary behavior, Sitting, Inactivity, Review, Cut-point, Recommendation

## Abstract

**Background:**

The appropriate limit to the amount of daily sedentary time (ST) required to minimize mortality is uncertain. This meta-analysis aimed to quantify the dose-response association between daily ST and all-cause mortality and to explore the cut-off point above which health is impaired in adults aged 18–64 years old. We also examined whether there are differences between studies using self-report ST and those with device-based ST.

**Methods:**

Prospective cohort studies providing effect estimates of daily ST (exposure) on all-cause mortality (outcome) were identified via MEDLINE, PubMed, Scopus, Web of Science, and Google Scholar databases until January 2018. Dose-response relationships between daily ST and all-cause mortality were examined using random-effects meta-regression models.

**Results:**

Based on the pooled data for more than 1 million participants from 19 studies, the results showed a log-linear dose-response association between daily ST and all-cause mortality. Overall, more time spent in sedentary behaviors is associated with increased mortality risks. However, the method of measuring ST moderated the association between daily ST and mortality risk (*p* < 0.05). The cut-off of daily ST in studies with self-report ST was 7 h/day in comparison with 9 h/day for those with device-based ST.

**Conclusions:**

Higher amounts of daily ST are log-linearly associated with increased risk of all-cause mortality in adults. On the basis of a limited number of studies using device-based measures, the findings suggest that it may be appropriate to encourage adults to engage in less sedentary behaviors, with fewer than 9 h a day being relevant for all-cause mortality.

**Electronic supplementary material:**

The online version of this article (10.1186/s12916-018-1062-2) contains supplementary material, which is available to authorized users.

## Background

A sedentary lifestyle is prevalent among adults in the present era. A recent multi-country study based on 12 sites in 10 countries including the USA, Brazil, the UK, Denmark, the Czech Republic, and China (Hong Kong) of adults aged 18–66 using accelerometry found that the average sedentary time (ST) per day was 8.65 h (standard deviation [SD] = 1.8) [1]. ST was estimated to be responsible for 3.8% of all-cause mortality in adults according to a meta-analysis pooling data across 54 countries [2]. Prolonged ST has been increasingly recognized as a serious issue in public health [3], and recommendations have begun to appear in public health guidelines [4], suggesting that all adults should minimize the amount of ST [5, 6]. To conduct screening and surveillance of the health hazards of a sedentary lifestyle and develop feasible intervention strategies and evidence-based recommendations, it is crucial to identify a cut-off or limit on the amount of ST per day, above which health is impaired.

The Australian government has proposed that that the cut-off point for risk is approximately 7 or 8 h a day [7], but the current evidence is inconsistent. Based on six studies (five using self-reported measures vs. one using a device-based measure), a meta-analysis examining the relationships between daily ST and all-cause mortality revealed that more than 7 h per day is associated with increased mortality risk [8]. In contrast, another recent meta-analysis based on 13 studies (all based on self-reported measures) found an increased risk of all-cause mortality among adults spending 4 or more hours per day in sedentary behaviors [9], which could be attenuated by the levels of moderate-to-vigorous physical activity (MVPA) as a moderator. Although the evident discrepancy may be due to heterogeneity across studies, one of the major limitations is that almost all the studies included in these two meta-analyses were based on self-report ST. Compared with devices, subjective measures such as questionnaires tend to be less accurate due to recall bias [10, 11]. Currently, there is insufficient evidence on which to provide specific public health recommendations regarding the appropriate limit to the amount of daily ST required to minimize mortality, especially using device-based assessments.

To address these shortfalls, our study involved meta-regression analyses to quantify the dose-response association between daily ST and all-cause mortality in adults aged 18–64 years old and to explore the cut-off duration associated with elevating the risk of all-cause mortality through reviewing evidence based on subjective measurements and recent studies using device-based ST [12–15]. We also examined whether there are distinct differences between studies involving self-report ST and those using device-based measures of ST.

## Methods

### Search strategy and selection criteria

Five databases, MEDLINE, PubMed, Scopus, Web of Science, and Google Scholar, were searched up to January 31, 2018 to identify potential studies examining relationships of sedentary behaviors with all-cause mortality in adults (aged 18–64 years). The following search strings were used: ((“sitting time” OR “sedentary behavior” OR “sedentary behavior”) AND (mortality OR mortalities OR death OR fatal)) AND (risk OR Cox OR hazard OR survival analysis OR odds). Additional studies were identified by manually checking the reference lists of included papers.

Article eligibility for inclusion was based on the following criteria: (1) original articles published in English before January 31, 2018; (2) articles involving a prospective cohort design; (3) involvement of participants in the age range of 18 to 64 years or the mean age in this range at baseline; (4) daily total ST or overall sitting time used as an exposure variable and all-cause mortality as an outcome variable; and (5) reported effect estimates of relative risk (RR) or odds ratios (ORs) or hazard ratios (HRs) with 95% confidence intervals (CIs) for all-cause mortality.

The exclusion criteria were applied to articles that: (1) focused on clinical populations such as patients with cardiovascular diseases, type 2 diabetes, or cancer etc.; (2) did not provide cut-off durations of total sedentary or sitting time; or (3) did not adjust for physical activity, since physical activity may be a confounding factor for the relationships of death with prolonged ST [12, 13].

### Data extraction and quality assessment

The following data were extracted from the retrieved articles: author(s), year of publication, country, study population (sample size/death, age at baseline, and gender), follow-up time, total ST measure, covariates that were adjusted for in the analysis, and the HR estimates with corresponding 95% CIs for the models. Two authors independently extracted the data from each study and compared them for consistency. Any discrepancies between the two reviewers were settled through discussion, and a third reviewer’s help was sought for resolving disagreements.

The study appraisal criteria and characteristics for each study are presented in Additional file 1: Table S1. Using the study quality checklist proposed by Kmet, Lee, and Cook [16], two authors (MH and YL) independently assessed the studies, and any disagreements were resolved by consensus. Studies were scored (0 for no, 1 for partial, 2 for yes) on 14 criteria by the following questions: Question/objective sufficiently described? and Study design evident and appropriate? [16], and the score of each study is presented in Additional file 2: Table S2. The sum of all scores was then divided by the highest possible score, giving quality scores ranging from 0 (worst) to 1 (best). A score ≥ 0.85 was defined as being of high quality [9].

### Statistical analysis

Categorization of ST was based on the data available from each study. The maximally adjusted HR estimates from multivariable proportional hazards models were utilized to reduce the confounding effect in each study. To identify the cut-off of ST duration for increasing the risk of all-cause mortality, “dose of ST” was assigned, using the median or mean level of ST in each category, to the corresponding relative risk for each study. When ST was reported by ranges of time, the midpoint of the range was estimated. When the highest category was open ended, the length of the open-ended interval was assumed to be the same as that of the adjacent interval. When the lowest category was open ended, the lower boundary was set to zero [17, 18]. Measures of association (HRs) and the corresponding CIs were transformed into the natural logarithm of the HRs and their variances. The statistical heterogeneity among studies was evaluated using *I*^*2*^ (i.e., the proportion of total variation contributed by between-study variance) [19].

To assess the shape of the associations of ST with log-transformed risk of all-cause mortality using pooled data extracted from the 19 prospective cohort studies, random-effects meta-regression models were used. Linear, quadratic, and cubic models were fitted to determine the model of best fit for the pooled dose-response data first [20]. Additionally, to explore a range of possible functions such as U-shaped and J-shaped patterns, second-order fractional polynomial models, including the quadratic model, were also comprehensively evaluated: (log HR │X) = β_1_X^P1^ + β_2_X^P2^. In this equation, P1 and P2 were chosen from a predefined set P = [− 2, − 1, − 0.5, 0, 0.5, 1, 2] [21]. The results of goodness-of-fit tests among these models (including the linear model, the second-order fractional polynomial models, and the cubic model) are shown in Additional file 3: Table S3. The model selection was based on two criteria: (1) more variance between studies were explained by the model (i.e., *R*^2^ analog) [22]; (2) the coefficients of each regression model were significantly different from zero. Among them, the linear model was chosen. Therefore, a random-effects meta-regression model based on linear dose-response relationships with restricted maximum likelihood estimations was utilized in the following analyses. To estimate the dispersion across studies and provide more accurate estimates, the Knapp-Hartung method was applied in the random-effects meta-regression analyses; this method additionally uses a refined estimator of between-studies variance of the effect estimator via a Student’s *t* distribution instead of a *Z* distribution [23, 24]. This method has the effect of expanding the width of the CIs and yields a more conservative inference.

Several random-effects meta-regression models were used as follows. First, the linear dose-response relationship between ST and all-cause mortality was examined based on all studies (Model 1). Second, the independent effects of ST and measurement of ST (device-based [1] vs. subjective [0]) on the heterogeneity of mortality risks were assessed in Model 2. Third, to assess whether measurement of ST moderates the association of ST with subsequent mortality risks across studies, Model 2 was rerun by further including an interaction term (ST × measurement of ST). Finally, given the statistically significant interaction effect (*p* < 0.05), two separate meta-regression models were conducted for studies using subjective measures and those with device-based instruments (Models 3 and 4).

Sensitivity analyses were performed to address potential confounding effects. The study-level variables, which may account for the heterogeneity of mortality risks, were scrutinized in a simple meta-regression model. In addition to measurement of ST (subjective vs. device-based), gender, mean age, year of publication, and mean length of follow-up were assessed. Among them, only mean length of follow-up reached significance (*p* < 0.05). Because of potential confounding due to the differences in length of study follow-up, the time for follow-up was further included in Model 2 (Model 5). Model 5 was also repeated by further including in it an interaction term (ST × measurement of ST).

To visualize the association of ST and mortality risk and identify the potential cut-off of ST, scatter plots with regression lines and 95% CIs (Model 2: total studies, Model 3: studies with self-reported ST, and Model 4: studies with device-based ST) were obtained using meta-regression models. The follow-up time of each study as a continuous variable was further included in the three models for adjustment.

Publication bias was evaluated by a visual investigation of funnel plots for potential asymmetry and assessed with Egger’s test [25] and Duval and Tweedie’s “trim and fill” test [26].

All analyses were performed with Comprehensive Meta-Analysis Version 3.3.070 (Biostat, Englewood, NJ, USA) [22]. All *p* values were two-sided and were considered significant at *p* < 0.05.

## Results

### Study characteristics

**Fig. 1:**
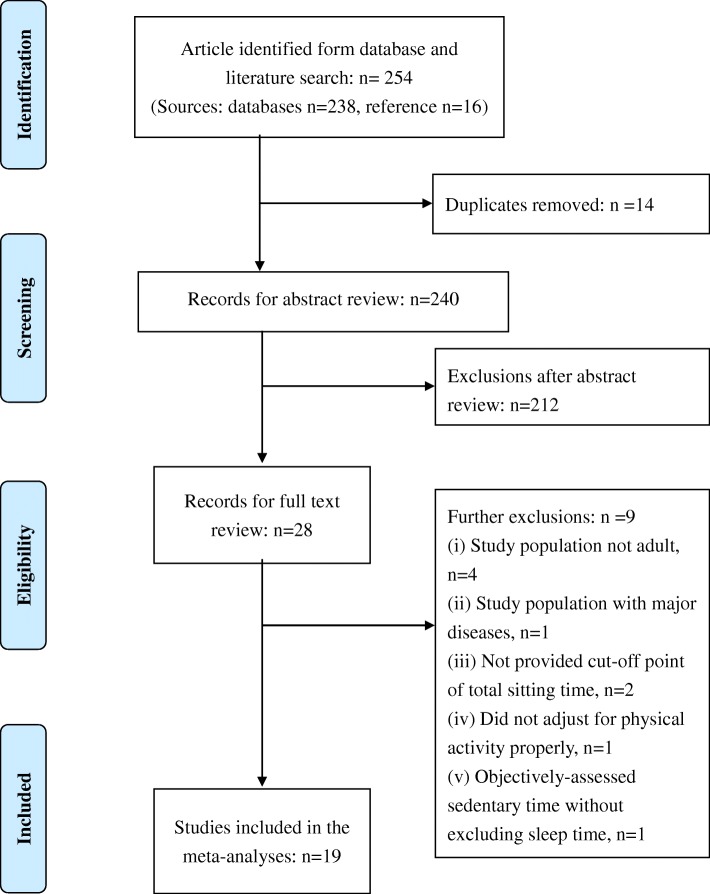
Flowchart of selection of studies for inclusion in meta-regression

A total of 254 articles were identified through five different database searches (*n* = 238) and reference list searches (*n* = 16) (see the Preferred Reporting Items for Systematic Reviews and Meta-Analyses [PRISMA] flowchart in Fig. 1) [27]. Subsequently, after duplicates were removed, a total of 240 articles were retrieved to endnote. When the abstracts were screened, a total of 28 full-text articles were obtained for further review. We removed 9 of these based on the following exclusion criteria after contacting the authors of the original studies when missing information was not available in their articles: (1) mean age of the study population was ≥ 65 (*n* = 4) [28–31]; (2) the study sample was based on participants in clinical trials on hormone therapy (*n* = 1) [32]; (3) the cut-off point of the total sitting time was not provided (*n* = 2) [33, 34]; (4) there was no adjustment for physical activity in the multivariable model (*n* = 1) [35]; (5) devices were used to estimate ST without excluding sleep time (*n* = 1) [36]. Finally, 19 studies were included for meta-analysis, and the quality scores were high in all studies (average = 0.96; ≥ 0.85 was defined as high quality) [9] (see Additional file 2: Table S2).

Data from all studies were extracted and are summarized in Additional file 1: Table S1. The 19 studies in the meta-analysis included 1,259,482 individuals who were followed up for 2.8–15.7 (mean time = 7.8) years, among whom 86,671 (6.9%) died [12–15, 37–47]. The mean age of participants in these studies ranged from 39.7 to 63.8 years old. Twelve studies assessed data by self-report ST [37–48] in comparison with seven studies with device-based ST [12–15, 49–51]. The measures of self-report ST among the 12 studies were brief. Seven studies utilized a single item [37–40, 42, 47, 48], three studies used two items [41, 45, 46], one used three items [44], and another one used five items [43]. The cut-off points for the categories were not consistent across the studies (see Additional file 1: Table S1). All studies adjusted for multiple potential confounding factors including at least gender, age, and physical activity, while 16 out of 19 studies also adjusted for education and smoking, 14 studies for body mass index (BMI), and 12 studies for alcohol consumption. Other covariates used for adjustment in the studies in this meta-analysis comprised race, marital status, urbanization, occupation, income, and comorbidity (see Additional file 1: Table S1).

The heterogeneity of effect estimates among studies based on *I*^2^ was 85.64%, suggesting a relatively high inconsistency across the findings in the included studies [52].

### Sedentary time and mortality: dose-response meta-regression

The meta-regression based on all included studies indicated a linear dose-response relationship between daily ST and log-transformed risk of all-cause mortality (Model 1 in Table 1). The Model 2 analyses demonstrated that both daily ST and measurement of ST (device-based vs. subjective) independently account for the heterogeneity in mortality risks. Model 2 was rerun after further inclusion of an interaction term, revealing a statistically significant interaction effect (*p* = 0.02).

**Table 1:** Dose-response relationships of sedentary time with all-cause mortality assessed using random-effects meta-regression models

Models	Number of ES	Coefficients (SE)	*t*	*p* value
Model 1	57			
Sedentary time		0.03 (0.01)	4.92	< 0.001
Model 2	57			
Sedentary time		0.03 (0.01)	5.08	< 0.001
Measurement (device-based = 1)		0.11 (0.05)	2.39	0.03
Model 3 (subjective measures)	36			
Sedentary time		0.03 (0.01)	5.09	< 0.001
Model 4 (device-based measures)	21			
Sedentary time		0.09 (0.03)	3.04	0.001
Model 5 (sensitivity analysis)	57			
Sedentary time		0.03 (0.005)	6.21	< 0.001
Measurement (device-based = 1)		0.09 (0.04)	2.19	0.03
Follow-up (5–9 vs. < 5 years)		−0.09 (0.04)	−2.16	0.04
Follow-up (10+ vs. < 5 years)		−0.16 (0.04)	−3.88	< 0.001

*ES* effect size, *SE* standard error

To test for moderation effects, the interaction term (sedentary time × measurement [device-based vs. subjective measure]) was further added into Model 2 (*p* = 0.02) and Model 5 (*p* = 0.01)

*t* Knapp-Hartung method

Two separate meta-regression models were then conducted for studies using subjective measures and those with device-based instruments (Models 3 and 4). ST was significantly associated with all-cause mortality in both models. However, the magnitude of associations was stronger in studies using devices (regression coefficient = 0.09) than in those based on subjective instruments (regression coefficient = 0.03).

In sensitivity analyses, we explored several study-level variables, such as gender, mean age, year of publication, and mean length of follow-up, which may account for the heterogeneity of mortality risks and possess potential confounding effects. Among them, only mean length of follow-up reached significance (*p* < 0.05), which was further included in Model 2 (Model 5). The results showed that studies with longer follow-up periods tended to have weaker associations between daily ST and mortality risks (see Table 1). The moderation effect of ST measurement was further examined in Model 5, indicating that the interaction effect remained similar (*p* = 0.01).

### Visual assessment of dose-response relationships

**Fig. 2:**
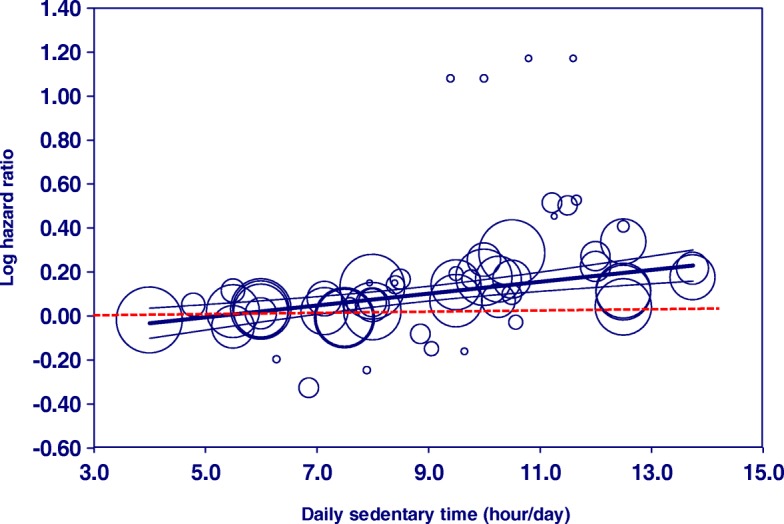
Meta-regression of all-cause mortality risk on daily sedentary time (including all studies). Each study is represented by a *circle*. The *size* of each circle is proportional to that study’s weight. The *center line* and the *upper and lower lines* show the predicted values and their 95% confidence intervals. Note: The meta-regression model was adjusted for follow-up time of each study

The scatter plot of Model 1 illustrates the association of log-transformed mortality risk and doses of sitting time per day treated as a continuous variable (Fig. 2). The regression line and the upper and lower lines for 95% CI showed that increased hazards of death from all causes became significant when total ST exceeded approximately 7.5 h/day.

**Fig. 3:**
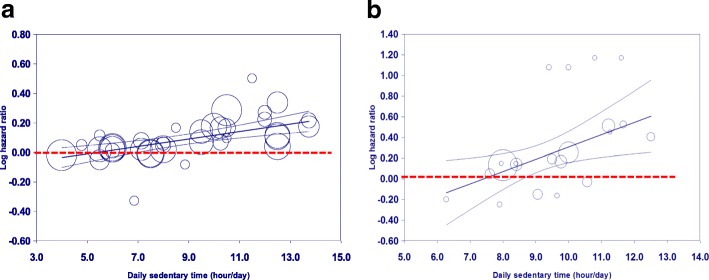
Meta-regression of all-cause mortality risk on daily sedentary time based on studies with different measures (**a** subjective vs. **b** device-based). Each study is represented by a *circle*. The *size* of each circle is proportional to that study’s weight. The *center line* and the *upper and lower lines* show the predicted values and their 95% confidence intervals. Note: The meta-regression models were adjusted for follow-up time of each study

The scatter plot of Model 3 (Fig. 3a) revealed that mortality risk significantly increased when daily ST exceeded 7 h/day in studies with subjective measurement. In contrast, the potential cut-off time duration for those with device-based assessment was close to 9 h (Fig. 3b).

### Assessment of publication bias

No evidence of funnel plot asymmetry was observed (Additional file 4: Figure S1). There was no indication of publication bias with Egger’s test, *p* = 0.46 or with the “trim and fill” adjustment. The observed point estimate in log units was 0.11 (95% CI 0.07–0.15), which is similar to the adjusted estimate after imputing two studies: 0.10 (95% CI 0.06–0.14).

## Discussion

The present meta-regression analyses based on pooled data for more than 1 million participants from 19 well-designed prospective cohort studies revealed a significant log-linear association between daily ST and all-cause mortality (i.e., HR) in adults. Overall, more time spent in sedentary behaviors is prospectively associated with increased mortality risks. Interestingly, there is a role for the method of measurement of ST in modulating the effect of daily ST on subsequent mortality risks across studies. The cut-off duration of daily ST in studies with subjective measures was more than 7 h. In contrast, the cut-off point for those with device-based measures was close to 9 h. These findings were supported by the meta-regression analyses adjusting for follow-up periods of each study. All of the pooled estimates were derived from large-scale prospective cohort studies with high-quality design and adjusted for multiple underlying confounding factors, including MVPA. Collectively, they provide additional evidence for ST recommendation.

The current meta-analysis study based on 19 prospective cohort studies (12 self-reported vs. 7 device-based) found that the optimal amount of daily ST in adults should be less than 7.5 h. This is close to a previous meta-analysis of cohort studies (5 self-reported vs. 1 device-based) [8], suggesting a cut-off time interval of 7 h, and is somewhat higher than the cut-off of 5 h (the midpoint of the category 4–6 h/day) revealed by another recent meta-analysis of cohort studies (13 studies all based on self-report measures) [9]. This inconsistency may be partly due to variation in the studies included in each review, which comprised studies based on different measures of ST.

This review using meta-regression found that the measurement method may moderate the associations between ST and all-cause mortality across studies. The magnitude of associations was stronger in studies using device-based devices than in those with self-report ST. Previous evidence suggests that questionnaires involving multiple contexts for assessing daily ST are more likely to overestimate total ST in comparison with accelerometer-based devices [53]. In contrast, daily ST assessed using a single item such as the International Physical Activity Questionnaire (IPAQ) leads to an underestimate of total daily ST ranging from 2 to 3.5 h [54, 55]. In the present review, 10 out of the 12 studies based on self-report ST employed only one or two items to assess daily ST. It is possible that a questionnaire with one or two items is not able to capture the variability of sedentary behaviors that occurs in different contexts. This may partially explain why the cut-off in studies with subjective measurement was 7 h/day in comparison with 9 h/day in those with device-based assessment, and why the magnitude of relationships was greater in studies using device-based measures. Therefore, the appropriate cut-off duration for daily ST in adults may be around 9 h, although this finding is based on a small number of studies with device-based measures. It is worth noting that the relationships of mortality risk (i.e., HR) with ST are log-linear. Participants spending more than 9 h/day had a significant increased risk of mortality (HR = 1.22), with a rapid escalation from 10 h/day (HR = 1.35), 12 h/day (HR = 1.63), to 14 h/day (HR = 1.96) (based on Model 1 in Table 1, data not shown).

The moderating effect of type of measurement on the relationships of ST with mortality risks was further supported by the sensitivity analysis that took the length of follow-up into account. Studies with longer follow-up periods were more likely to have weaker associations between daily ST and mortality risks. This issue has not been documented in previous relevant meta-analyses [8, 9], and there is no clear explanation for the result. But it is possible that sedentary behaviors change over time, attenuating the associations between baseline estimates and all-cause mortality. Although the studies with a shortened period of follow-up may increase the possibility of reverse causality, several studies included in this review have demonstrated that similar results remained after excluding those dying in the first year [15, 40, 47] or in the first 3 years [42].

There are several strengths in this meta-analysis. First, it is the first meta-regression based on 19 high-quality cohort studies that has examined the moderating effect of type of ST measurement on the dose-response relationships with mortality risk. Second, the large-scale pooled data for more than 1 million participants allowed the dose-response analyses to yield more precise effect estimates than previously obtained. Finally, mortality ascertainment was based on official death registry records, which is more likely to be accurate than other methods of assessment.

The main limitation of this meta-analysis is the small number of high-quality studies, especially those with device-based ST [8]. Furthermore, although the pooled estimates were based on large-scale prospective cohort studies with high-quality design and adjusted for multiple underlying confounding factors including moderate-to-vigorous physical activity (MVPA), there remains the possibility of reverse causality or unmeasured confounding [8]. The mean age of participants in the studies analyzed ranged from 39.7 to 63.8 years old, which may limit the generalizability of the findings to the wider adult population. Additionally, the studies using device-based measures in the current review provide more accuracy of ST estimation, but they could not detect the difference between standing and sitting, which is a limitation of monitoring daily sedentary time. Finally, the current analyses were based on all-cause mortality as the outcome, and other thresholds for ST duration may be relevant to different outcomes, such as non-fatal illness or adiposity.

An international study involving 10 countries using accelerometry found that average sedentary time (ST) per day was 8.65 h among adults [1], which is close to the cut-off (9 h) of daily ST in adults observed in the current study. This means that nearly half of adults are at risk of increased mortality, and immediate action is needed to address the rise of sedentary lifestyle as a global trend. A previous meta-analysis demonstrated that MVPA potentially moderates the association of ST with mortality. Those who were active for about 60–75 min of MVPA every day did not have an increased risk of mortality even if they sat for more than 8 h per day [9]. Notably, those findings indicated distinct sitting-mortality effects at different levels of MVPA, revealing that the cut-off of ST may be different among adults with different levels of MVPA. However, those meta-analyses were all based on studies using self-reported measures of ST, which should be further verified using studies with device-based ST, especially with a large sample size.

## Conclusions

This meta-analysis suggests that there is a log-linear dose-response association between daily ST and all-cause mortality in adults. The method of measurement could moderate the relationships of daily ST with subsequent mortality risks. This review suggests that it is appropriate to encourage adults to engage in less sedentary behaviors, with fewer than 9 h a day being relevant for all-cause mortality. There is a pressing need for more longitudinal studies involving device-based measures of ST and examining other thresholds for ST duration for all-cause mortality and other different outcomes such as non-fatal illness or adiposity.

## Additional files

Additional file 1: **Table S1.** Characteristics of studies included in the meta-regression. (DOCX 37 kb)

Additional file 2: **Table S2.** Quality assessment of systematic reviews by Kmet, Lee, and Cook rating. (DOCX 27 kb)

Additional file 3: **Table S3.** Goodness of fit for meta-regression models. (DOCX 22 kb)

Additional file 4: **Figure S1.** Funnel plot of standard error by log rate ratio. (PDF 7 kb)

## Abbreviations

BMI: Body mass index
CI: Confidence interval
HR: Hazard ratio
M: Mean
MVPA: Moderate to vigorous physical activity
OR: Odds ratio
PA: Physical activity
RR: Relative risk
SE: Standard error
ST: Sedentary time
