# Supplementary material for: A cut-off of daily sedentary time and all-cause mortality in adults: a meta-regression analysis involving more than 1 million participants
Source: BMC Med. 2018 May 25;16:74. doi: 10.1186/s12916-018-1062-2 (PMC5998593; doi:10.1186/s12916-018-1062-2)
Supplement: Supplementary file 1 — Table S1. Characteristics of studies included in the meta-regression. (DOCX 37 kb) [file 12916_2018_1062_MOESM1_ESM.docx]

**Table S1** Characteristics of studies included in the meta-regression

| Author, year,  Country | Study population | | | Follow-up (year) | SB measure  (number of items) | Covariates | Cut-off (h/d) | Cox regression  HR (95% CI) | Quality  assessment |
| --- | --- | --- | --- | --- | --- | --- | --- | --- | --- |
|  | n/Death | Age | Gender |  |  |  |  |  |  |
| Inoue et al., 2008, Japan | 83034/  4564 | 45–74  *M*=56.2 | Female  52.8% | 8.7y | Self-report sedentary activity time (1) | Age, area, occupation, BMI, history of diabetes, smoking, alcohol, total energy intake, heavy physical work or strenuous exercise, walking or standing hours, leisure-time sports or physical exercise |  | Male | 0.95 |
|  |  |  |  |  |  |  | <3 (ref.) | 1.00 ^a^ |  |
|  |  |  |  |  |  |  | 3–<8 | 1.02 (0.95–1.11) |  |
|  |  |  |  |  |  |  | ≥8 | 1.18 (1.04–1.35)^*^ |  |
|  |  |  |  |  |  |  |  | Female |  |
|  |  |  |  |  |  |  | <3 (ref.) | 1.00 ^b^ |  |
|  |  |  |  |  |  |  | 3–<8 | 0.95 (0.85–1.06) |  |
|  |  |  |  |  |  |  | ≥8 | 1.10 (0.82–1.25) |  |
| Koster et al., 2012, US | 1906/  145 | ≥50  *M*=63.8 (10.5) | Female  54% | 2.8y | objectively measured SB <100 counts/1min [AM-7164 ActiGraph] | Age, gender, race/ethnicity, education, BMI, diabetes, coronary heart disease, congestive heart failure, cancer, stroke, mobility limitation, smoking, alcohol, MVPA | Quartile: male/ female | | 1.0 |
|  |  |  |  |  |  |  | <7.6/<7.0 (ref.) | 1.00 ^c^ |  |
|  |  |  |  |  |  |  | 7.6-9.1 / 7.2-8.6 | 1.16 (0.48–2.83) |  |
|  |  |  |  |  |  |  | 9.2-10.7 / 8.7-10.0 | 2.94 (1.42–6.08)^*^ |  |
|  |  |  |  |  |  |  | 10.8 / 10.1 | 3.22 (1.39–7.44)^*^ |  |
| Matthews et al., 2012, US | 240819/  17044 | 50–71  *M*=62.1 | ? ^e^ | 8.5y | Self-report sitting time (1) | Age, sex, race, education, smoking, diet quality, MVPA | 0<3 (ref.) | 1.00 ^a^ | 0.93 |
|  |  |  |  |  |  |  | 3–< 5 | 0.98 (0.95–1.03) |  |
|  |  |  |  |  |  |  | 5–< 7 | 1.03 (0.98–1.08) |  |
|  |  |  |  |  |  |  | 7–< 9 | 1.02 (0.96–1.07) |  |
|  |  |  |  |  |  |  | ≥9 | 1.19 (1.12–1.27)^*^ |  |

| van der Ploeg et al., 2012, Australia | | 222497/  5405 | | ≥45  *M*=62.7^d^ | | Female 52.4% | | 2.8y | | Self-report sitting time (1) | | Age, sex education, marital status, urbanization, BMI, self-rated health, receiving help with daily tasks for long-term illness or disability, smoking, and PA | | 0 –<4 (ref.) | | 1.00 ^a^ | | 0.98 | |
| --- | --- | --- | --- | --- | --- | --- | --- | --- | --- | --- | --- | --- | --- | --- | --- | --- | --- | --- | --- |
|  |  |  |  |  |  |  |  |  |  |  |  |  |  | 4 – <8 | | 1.02 (0.95–1.09) | |  |  |
|  |  |  |  |  |  |  |  |  |  |  |  |  |  | 8 – <11 | | 1.15 (1.06–1.25)^*^ | |  |  |
|  |  |  |  |  |  |  |  |  |  |  |  |  |  | ≥11 | | 1.40 (1.27–1.55)^*^ | |  |  |
| Kim et al., 2013, US | | 134596/  19143 | | 45–75  *M*(male)=58.7  *M*(female)=58.5 | | Female 54.4% | | 13.7y | | Self-report sitting activities: sitting in a car or bus, work, meals, TV and other leisure sitting activities, e.g., reading, playing cards, sewing. (5) | | Age, ethnicity, education, history of hypertension and/or diabetes at enrolment, smoking, alcohol, energy intake, PA | |  | | Male | | 0.95 | |
|  |  |  |  |  |  |  |  |  |  |  |  |  |  | <5 (ref.) | | 1.00 ^b^ | |  |  |
|  |  |  |  |  |  |  |  |  |  |  |  |  |  | 5–<10 | | 0.99 (0.94–1.04) | |  |  |
|  |  |  |  |  |  |  |  |  |  |  |  |  |  | ≥10 | | 1.04 (0.98–1.11) | |  |  |
|  |  |  |  |  |  |  |  |  |  |  |  |  |  |  | | Female | |  |  |
|  |  |  |  |  |  |  |  |  |  |  |  |  |  | <5 (ref.) | | 1.00 ^a^ | |  |  |
|  |  |  |  |  |  |  |  |  |  |  |  |  |  | 5–<10 | | 0.99 (0.93–1.05) | |  |  |
|  |  |  |  |  |  |  |  |  |  |  |  |  |  | ≥10 | | 1.11 (1.04–1.19)^*^ | |  |  |
| Bjørk Petersen et al., 2014, Denmark | | 71363/  1074 | | 18–99  *M*=48.1 (15.3) | | Female 60.5% | | 5.4y | | Self-report sitting during work and leisure time on: a weekday and a weekend day (2) | | Age, sex, education, BMI, diabetes, hypertension, smoking, alcohol, PA level in leisure time | | 0–< 6 (ref.) | | 1.00 ^a^ | | 0.98 | |
|  |  |  |  |  |  |  |  |  |  |  |  |  |  | 6– < 10 | | 1.05 (0.90–1.22) | |  |  |
|  |  |  |  |  |  |  |  |  |  |  |  |  |  | ≥10 | | 1.31 (1.09–1.57)^*^ | |  |  |
| Matthews et al., 2014, US | | 63308/  3613 (black),  1394  (white) | | 40–79  *M*=51.3 | | ? ^e^ | | 6.4y | | Self-report sitting time in a car or bus, work, watching TV or movies, using computer at home, and doing other sitting activities, e.g., eating meals, talking on the phone, reading, playing cards, or sewing. (1) | | Age, sex, race, education, household income, marital status, occupational status, BMI, diabetes, smoking, sleep duration, PA | |  | | Black | | 0.95 | |
|  |  |  |  |  |  |  |  |  |  |  |  |  |  | <5.76 (ref.) | | 1.00 ^a^ | |  |  |
|  |  |  |  |  |  |  |  |  |  |  |  |  |  | 5.76–<8.51 | | 1.02 (0.93–1.12) | |  |  |
|  |  |  |  |  |  |  |  |  |  |  |  |  |  | 8.51–<12.00 | | 1.18 (1.07–1.30)^*^ | |  |  |
|  |  |  |  |  |  |  |  |  |  |  |  |  |  | ≥12.00 | | 1.19 (1.08–1.32)^*^ | |  |  |
|  |  |  |  |  |  |  |  |  |  |  |  |  |  |  | | White ^a^ | |  |  |
|  |  |  |  |  |  |  |  |  |  |  |  |  |  | <5.76 (ref.) | | 1.00 | |  |  |
|  |  |  |  |  |  |  |  |  |  |  |  |  |  | 5.76–<8.51 | | 1.08 (0.93, 1.25) | |  |  |
|  |  |  |  |  |  |  |  |  |  |  |  |  |  | 8.51–<12.00 | | 1.07 (0.92, 1.25) | |  |  |
|  |  |  |  |  |  |  |  |  |  |  |  |  |  | ≥12.00 | | 1.24 (1.06, 1.47)^*^ | |  |  |

| Seguin et al., 2014, US | | 92234/  13316 | | 50–79  *M*=63.6 (7.4) | | Female | | 12.2y | | Self-report sitting time: work, eating, driving or riding in a car or bus, and watching TV or talking; lying down: sleeping or trying to sleep at night, resting or napping, and lying down watching TV. (2) | | Age, race, education, marital status, living alone, BMI, self-rated health status, falls, hormone use, chronic diseases, depressed mood, activity of daily living disability, history of coronary heart disease, congestive heart failure, history of stroke, treated diabetes, hypertensive, arthritis, cancer, chronic obstructive pulmonary disease, and history of hip fracture over age 55, physical functioning, smoking, alcohol, PA | | ≤4 (ref.) | | 1.00 ^a^ | | 0.95 | |
| --- | --- | --- | --- | --- | --- | --- | --- | --- | --- | --- | --- | --- | --- | --- | --- | --- | --- | --- | --- |
|  |  |  |  |  |  |  |  |  |  |  |  |  |  | 4–< 8 | | 1.03 (0.97–1.10) | |  |  |
|  |  |  |  |  |  |  |  |  |  |  |  |  |  | 8–< 11 | | 1.07 (0.99–1.14) | |  |  |
|  |  |  |  |  |  |  |  |  |  |  |  |  |  | ≥11 | | 1.12 (1.05–1.21)^*^ | |  |  |
| Chau et al., 2015, Norway | | 50817/  640 | | ≥20  *M*=50.9 | | Female  54.6% | | 3.3y | | Self-report sitting time, include work hours and leisure time. (1) | | Age, sex, education, BMI, general health status, cardiometabolic diseases with age as the time axis, smoking, PA | | <4(ref.) | | 1.00 ^a^ | | 0.98 | |
|  |  |  |  |  |  |  |  |  |  |  |  |  |  | 4–<7 | | 1.12(0.89–1.42) | |  |  |
|  |  |  |  |  |  |  |  |  |  |  |  |  |  | 7–<10 | | 1.18 (0.90–1.57) | |  |  |
|  |  |  |  |  |  |  |  |  |  |  |  |  |  | ≥10 | | 1.65 (1.24–2.21)^*^ | |  |  |
| Ding et al., 2015, Australian | | 231048/  15635 | | 45–≥80  *M*=62.2^d^ | | Female  52.9% | | 6.1y | | Self-report sitting time (1) | | Age, sex, education, marital status, country of birth, area of residence, cardiovascular, metabolic disease, cancer, BMI, smoking, alcohol, dietary behavior, sleep duration, PA | | <7 (ref.) | | 1.00 ^c^ | | 0.9 | |
|  |  |  |  |  |  |  |  |  |  |  |  |  |  | ≥7 | | 1.33 (1.29–1.38)^*^ | |  |  |
| Pulsford et al., 2015, UK | | 5132/  450 | | 35–55  *M*=43.9^d^ | | Female  27.5% | | 15.7y | | Self-report sitting time: work sitting (including commuting), TV viewing time, non-TV leisure time sitting. (3) | | Age, gender, ethnicity, employment, BMI, physical functioning, smoking, alcohol, fruit and vegetable, daily walking time, MVPA | | <3.71 (ref.) | | 1.00^b^ | | 0.93 | |
|  |  |  |  |  |  |  |  |  |  |  |  |  |  | 3.71–<5.86 | | 1.05 (0.83–1.33) | |  |  |
|  |  |  |  |  |  |  |  |  |  |  |  |  |  | 5.86–<7.86 | | 0.72 (0.54–0.98) | |  |  |
|  |  |  |  |  |  |  |  |  |  |  |  |  |  | ≥7.86 | | 0.92 (0.69–1.22) | |  |  |

| Edwards et al., 2016, US | | 2295/  101 | | 20-85  *M*=39.7 | | Female  50.7% | | 6.8y | | objectively measured SB <100 counts/1min [AM-7164 ActiGraph] | | Age, gender, race/ethnicity, income, Cardiorespiratory fitness, MVPA | | <7.77 (ref.) | | 1.00 ^b^ | | 1.0 | |
| --- | --- | --- | --- | --- | --- | --- | --- | --- | --- | --- | --- | --- | --- | --- | --- | --- | --- | --- | --- |
|  |  |  |  |  |  |  |  |  |  |  |  |  |  | ≥7.77 | | 1.69 (0.96–2.94) | |  |  |
| Evenson et al., 2016, US | | 3809/325 | | ≥40  *M*=55.3 | | Female  54.6% | | 6.7y | | objectively measured SB <100 counts/1min [AM-7164 ActiGraph] | | Age, sex, race/ethnicity, education, married, interaction between current, employment, follow-up time, need special equipment to walk, arthritis, cancer, BMI, interaction between BMI categories and follow-up time, hypertension, diabetes, smoking, light PA, MVPA | | <6.89 (ref.) | | 1.00^b^ | | 0.95 | |
|  |  |  |  |  |  |  |  |  |  |  |  |  |  | 6.89–<8.30 | | 1.05 (0.71–1.55) | |  |  |
|  |  |  |  |  |  |  |  |  |  |  |  |  |  | 8.30–<9.81 | | 0.86 (0.58–1.27) | |  |  |
|  |  |  |  |  |  |  |  |  |  |  |  |  |  | ≥9.81 | | 0.97 (0.65–1.44) | |  |  |
| Hagger-Johnson et al., 2016, US | | 12778/  577 | | 37–78  *M*=55.6 (8.8) | | Female | | 12y | | Self-report sitting time: a weekday and a weekend day (2) | | Age, sex, education, occupational, retirement status, chronic disease, smoking, alcohol, fruit/vegetable consumption, sleep hours, PA | | <5(ref.) | | 1.00 ^c^ | | 0.95 | |
|  |  |  |  |  |  |  |  |  |  |  |  |  |  | 5–< 7 | | 1.01 (0.85–1.19) | |  |  |
|  |  |  |  |  |  |  |  |  |  |  |  |  |  | ≥7 | | 1.06 (0.88–1.29) | |  |  |
| Lee, 2016, US | | 7006/608 | | ≥18  *M*=48^d^ | | Female 51.5% | | 6.7y | | objectively measured SB <100 counts/1min [AM-7164 ActiGraph] | | Age, sex, education, income, BMI, self-reported general health, condition, high blood pressure, high cholesterol, type 2 diabetes, and history of heart attack, stroke, and cancer, energy intake by 24-h dietary recall, binge drinking, smoking, MVPA | | < 7.76 (ref.) | | 1.00 (ref.)^c^ | | 0.95 | |
|  |  |  |  |  |  |  |  |  |  |  |  |  |  | 7.77–<9.05 | | 1.15 (0.84–1.57) | |  |  |
|  |  |  |  |  |  |  |  |  |  |  |  |  |  | 9.05–<10.50 | | 1.18 (0.87–1.59) | |  |  |
|  |  |  |  |  |  |  |  |  |  |  |  |  |  | ≥10.50 | | 1.67 (1.26–2.21)^*^ | |  |  |
| Matthews et al., 2016, US | | 4840/  700 | | ≥40  *M*=56.8 | | Female  50.3% | | 6.6y | | objectively measured SB <100 counts/1min [AM-7164 ActiGraph] | | Age, sex, race, education, BMI, diabetes, coronary artery disease, cancer, stroke, mobility limitations, smoking, alcohol, MVPA | | 6 (ref.)^+^ | | 1.00 ^c^ | | 1.0 | |
|  |  |  |  |  |  |  |  |  |  |  |  |  |  | 8 | | 1.14 (1.1–1.2)^*^ | |  |  |
|  |  |  |  |  |  |  |  |  |  |  |  |  |  | 10 | | 1.29 (1.1–1.5)^*^ | |  |  |

| Evenson et al., 2017, US | | 4510/  513 | | ≥40  *M*= 56.5 | | Female  53.7% | | 6.6y | | objectively measured SB <100 counts/1min [AM-7164 ActiGraph] | | Age, gender, race, education, married, employment, need special equipment to walk, arthritis, cancer, angina, myocardial infarction, stroke, coronary heart disease, congestive heart failure, hypertension, diabetes, BMI, smoking, light and MVPA | | 4.54 (ref.)^+^ | | 1.0^a^ | | 0.95 | |
| --- | --- | --- | --- | --- | --- | --- | --- | --- | --- | --- | --- | --- | --- | --- | --- | --- | --- | --- | --- |
|  |  |  |  |  |  |  |  |  |  |  |  |  |  | 6.28 | | 0.82 (0.39–1.71) | |  |  |
|  |  |  |  |  |  |  |  |  |  |  |  |  |  | 7.90 | | 0.78 (0.37–1.62) | |  |  |
|  |  |  |  |  |  |  |  |  |  |  |  |  |  | 9.65 | | 0.85 (0.38–1.90) | |  |  |
|  |  |  |  |  |  |  |  |  |  |  |  |  |  | 11.26 | | 1.57 (0.64–3.85) | |  |  |
| Grunseit et al., 2017, Norway | | 25651/  1212 | | ≥20  *M*=45.2^d^ | | Female  52.6% | | 6.2y | | Self-report sitting time. (1) | | Age, sex, education, BMI, general health status, cardiometabolic diseases, smoking, light PA, hard PA | | <8 (ref.) | | 1.00^c^ | | 0.9 | |
|  |  |  |  |  |  |  |  |  |  |  |  |  |  | ≥8 | | 1.25 (1.05–1.49)^*^ | |  |  |
| Koolhaas et al., 2017, Netherlands | | 1839/  212 | | 45-98  *M*=63 | | Female  54.6% | | 11y | | objectively measured SB <199 counts/1min [Actiwatch model AW4] | | Age, sex, education, number of comorbidities, the 24h activity rhythm, activities of daily living score, cohort and time awake, smoking, alcohol, PA | | <8 (ref.) | | 1.00 ^b^ | | 1.0 | |
|  |  |  |  |  |  |  |  |  |  |  |  |  |  | 8–<11 | | 1.21 (0.81–1.81) | |  |  |
|  |  |  |  |  |  |  |  |  |  |  |  |  |  | ≥11 | | 1.50 (0.93–2.14) | |  |  |
| Total n=1,259,482/  Deceased n= 86,671 | | | | M=55.1  (±7.2)y | |  | | *M*=7.8  (±3.6)y | |  | |  | |  | |  | | *M*=0.96 | |

^*^*p*= <.05

Abbreviations: *M*: mean, HR: hazard ratios, PA: physical activity, MVPA: moderate to vigorous physical activity.

Tests for linear trend: a (significant, *p* < 0.05), b (non-significant), c (not reported); d: Five studies did not report mean age of the study samples. The mean age of these studies were recalculated as follows: ∑(median age of a age group)🞨 (sample size of a age group)] divided by the total sample size; e: Two studies did not report sex percentage of the study samples.

+: The mean level of sedentary time in each category
