# Supplementary material for: A cut-off of daily sedentary time and all-cause mortality in adults: a meta-regression analysis involving more than 1 million participants
Source: BMC Med. 2018 May 25;16:74. doi: 10.1186/s12916-018-1062-2 (PMC5998593; doi:10.1186/s12916-018-1062-2)
Supplement: Supplementary file 2 — Table S2. Quality assessment of systematic reviews by Kmet, Lee, and Cook rating. (DOCX 27 kb) [file 12916_2018_1062_MOESM2_ESM.docx]

**Table S2** Quality assessment of systematic reviews by Kmet, Lee and Cook rating

| Author (Year) | Assessment items | | | | | | | | | | | | | | |
| --- | --- | --- | --- | --- | --- | --- | --- | --- | --- | --- | --- | --- | --- | --- | --- |
|  | 1 | 2 | 3 | 4 | 5 | 6 | 7 | 8 | 9 | 10 | 11 | 12 | 13 | 14 | Overall rating |
| Inoue et al., 2008 | Yes | Yes | Yes | Yes | N/A | N/A | N/A | Partial | Yes | Yes | N/A | Yes | Yes | Yes | 0.95 |
|  | Yes | Yes | Yes | Yes | N/A | N/A | N/A | Partial | Yes | Yes | N/A | Yes | Yes | Yes |  |
| Koster et al., 2012 | Yes | Yes | Yes | Yes | N/A | N/A | N/A | Yes | Yes | Yes | N/A | Yes | Yes | Yes | 1.0 |
|  | Yes | Yes | Yes | Yes | N/A | N/A | N/A | Yes | Yes | Yes | N/A | Yes | Yes | Yes |  |
| Matthews et al., 2012 | Yes | Yes | Yes | Yes | N/A | N/A | N/A | Partial | Yes | Yes | N/A | Yes | Yes | Yes | 0.93 |
|  | Yes | Yes | Yes | Yes | N/A | N/A | N/A | Partial | Yes | Yes | N/A | Partial | Yes | Yes |  |
| van der Ploeg et al., 2012 | Yes | Yes | Yes | Yes | N/A | N/A | N/A | Yes | Yes | Yes | N/A | Yes | Yes | Yes | 0.98 |
|  | Yes | Yes | Yes | Yes | N/A | N/A | N/A | Yes | Yes | Yes | N/A | Yes | Yes | Yes |  |
| Kim et al., 2013 | Yes | Yes | Yes | Yes | N/A | N/A | N/A | Partial | Yes | Yes | N/A | Yes | Yes | Yes | 0.95 |
|  | Yes | Yes | Yes | Yes | N/A | N/A | N/A | Partial | Yes | Yes | N/A | Yes | Yes | Yes |  |
| Bjørk Petersen et al., 2014 | Yes | Yes | Yes | Yes | N/A | N/A | N/A | Yes | Yes | Yes | N/A | Yes | Yes | Yes | 0.98 |
|  | Yes | Yes | Yes | Yes | N/A | N/A | N/A | Partial | Yes | Yes | N/A | Yes | Yes | Yes |  |
| Matthews et al., 2014 | Yes | Yes | Yes | Yes | N/A | N/A | N/A | Partial | Yes | Yes | N/A | Yes | Yes | Yes | 0.95 |
|  | Yes | Yes | Yes | Yes | N/A | N/A | N/A | Partial | Yes | Yes | N/A | Yes | Yes | Yes |  |
| Seguin et al., 2014 | Yes | Yes | Yes | Yes | N/A | N/A | N/A | Partial | Yes | Yes | N/A | Yes | Yes | Yes | 0.95 |
|  | Yes | Yes | Yes | Yes | N/A | N/A | N/A | Partial | Yes | Yes | N/A | Yes | Yes | Yes |  |
| Chau et al., 2015 | Yes | Yes | Yes | Yes | N/A | N/A | N/A | Yes | Yes | Yes | N/A | Yes | Yes | Yes | 0.98 |
|  | Yes | Yes | Yes | Yes | N/A | N/A | N/A | Partial | Yes | Yes | N/A | Yes | Yes | Yes |  |
| Ding et al., 2015 | Yes | Yes | Yes | Yes | N/A | N/A | N/A | Partial | Yes | Yes | N/A | Partial | Yes | Yes | 0.9 |
|  | Yes | Yes | Yes | Yes | N/A | N/A | N/A | Partial | Yes | Yes | N/A | Partial | Yes | Yes |  |
| Pulsford et al., 2015 | Yes | Yes | Yes | Yes | N/A | N/A | N/A | Partial | Yes | Yes | N/A | Yes | Yes | Yes | 0.93 |
|  | Yes | Yes | Yes | Yes | N/A | N/A | N/A | Partial | Yes | Partial | N/A | Yes | Yes | Yes |  |
| Edwards et al., 2016 | Yes | Yes | Yes | Yes | N/A | N/A | N/A | Yes | Yes | Yes | N/A | Yes | Yes | Yes | 1.0 |
|  | Yes | Yes | Yes | Yes | N/A | N/A | N/A | Yes | Yes | Yes | N/A | Yes | Yes | Yes |  |
| Evenson et al., 2016 | Yes | Yes | Yes | Yes | N/A | N/A | N/A | Yes | Yes | Yes | N/A | Partial | Yes | Yes | 0.95 |
|  | Yes | Yes | Yes | Yes | N/A | N/A | N/A | Yes | Yes | Yes | N/A | Partial | Yes | Yes |  |
| Hagger-Johnson et al., 2016 | Yes | Yes | Yes | Yes | N/A | N/A | N/A | Partial | Yes | Yes | N/A | Yes | Yes | Yes | 0.95 |
|  | Yes | Yes | Yes | Yes | N/A | N/A | N/A | Partial | Yes | Yes | N/A | Yes | Yes | Yes |  |
| Lee, 2016 | Yes | Yes | Yes | Yes | N/A | N/A | N/A | Yes | Yes | Yes | N/A | Partial | Partial | Yes | 0.95 |
|  | Yes | Yes | Yes | Yes | N/A | N/A | N/A | Yes | Yes | Yes | N/A | Partial | Yes | Yes |  |
| Matthews et al., 2016 | Yes | Yes | Yes | Yes | N/A | N/A | N/A | Yes | Yes | Yes | N/A | Yes | Yes | Yes | 1.0 |
|  | Yes | Yes | Yes | Yes | N/A | N/A | N/A | Yes | Yes | Yes | N/A | Yes | Yes | Yes |  |
| Evenson et al., 2017 | Yes | Yes | Yes | Yes | N/A | N/A | N/A | Yes | Yes | Yes | N/A | Partial | Yes | Yes | 0.95 |
|  | Yes | Yes | Yes | Yes | N/A | N/A | N/A | Yes | Yes | Yes | N/A | Partial | Yes | Yes |  |
| Grunseit et al., 2017 | Yes | Yes | Yes | Yes | N/A | N/A | N/A | Partial | Yes | Yes | N/A | Partial | Yes | Yes | 0.9 |
|  | Yes | Yes | Yes | Yes | N/A | N/A | N/A | Partial | Yes | Yes | N/A | Partial | Yes | Yes |  |
| Koolhaas et al., 2017 | Yes | Yes | Yes | Yes | N/A | N/A | N/A | Yes | Yes | Yes | N/A | Yes | Yes | Yes | 1.0 |
|  | Yes | Yes | Yes | Yes | N/A | N/A | N/A | Yes | Yes | Yes | N/A | Yes | Yes | Yes |  |

All items were scored as “Yes (2)”, “Partial (1)”, “No (0)”, “N/A=Not Applicable”. The quality checklist comprises the following items: 1. Question/objective sufficiently described; 2. Study design evident and appropriate; 3. Method of subject/comparison group selection of information/input variables described and appropriate; 4. Subject (and comparison group, if applicable) characteristics sufficiently described; 5. If interventional and random allocation was possible, was it described; 6.If interventional and blinding of investigators was possible, was it reported; 7. If interventional and blinding of subjects was possible, was it reported; 8. Outcome and (if applicable) exposure measure(s) well defined and robust to measurement / misclassification bias? means of assessment reported; 9. Sample size appropriate; 10. Analytic methods described/justified and appropriate; 11. Some estimate of variance is reported for the main results; 12. Controlled for confounding; 13. Result reported in sufficient detail; 14. Conclusions supported by the results?
