# Supplementary material for: A cut-off of daily sedentary time and all-cause mortality in adults: a meta-regression analysis involving more than 1 million participants
Source: BMC Med. 2018 May 25;16:74. doi: 10.1186/s12916-018-1062-2 (PMC5998593; doi:10.1186/s12916-018-1062-2)
Supplement: Supplementary file 3 — Table S3. Goodness of fit for meta-regression models. (DOCX 22 kb) [file 12916_2018_1062_MOESM3_ESM.docx]

| Models | P-values of regression coefficients | | | | | | R^2^ analog. |
| --- | --- | --- | --- | --- | --- | --- | --- |
|  | P1 | *P*-value | P2 | *P*-value | P3 | P-value |  |
| Linear |  |  |  |  |  |  |  |
|  | 1 | < 0.001 |  |  |  |  | 0.54 |
| 2nd-order fractional polynomials | | |  |  |  |  |  |
|  | -2 | 0.54 | -2 | 0.10 |  |  | 0.51 |
|  | -2 | 0.52 | -1 | 0.07 |  |  | 0.45 |
|  | -2 | 0.47 | -0.5 | 0.03 |  |  | 0.50 |
|  | -2 | 0.58 | 0 | 0.02 |  |  | 0.51 |
|  | -2 | 0.83 | 0.5 | 0.01 |  |  | 0.51 |
|  | -2 | 0.89 | 1 | 0.01 |  |  | 0.51 |
|  | -2 | 0.40 | 2 | 0.02 |  |  | 0.50 |
|  | -1 | 0.22 | -1 | 0.03 |  |  | 0.57 |
|  | -1 | 0.27 | -0.5 | 0.10 |  |  | 0.53 |
|  | -1 | 0.20 | 0 | 0.04 |  |  | 0.54 |
|  | -1 | 0.45 | 0.5 | 0.06 |  |  | 0.52 |
|  | -1 | 0.79 | 1 | 0.08 |  |  | 0.51 |
|  | -1 | 0.54 | 2 | 0.14 |  |  | 0.49 |
|  | -0.5 | <0.001 | -0.5 | 0.22 |  |  | 0.50 |
|  | -0.5 | 0.58 | 0 | 0.16 |  |  | 0.52 |
|  | -0.5 | 0.79 | 0.5 | 0.19 |  |  | 0.51 |
|  | -0.5 | 0.79 | 1 | 0.24 |  |  | 0.51 |
|  | -0.5 | 0.26 | 2 | 0.35 |  |  | 0.50 |
|  | 0 | 0.62 | 0 | 0.37 |  |  | 0.52 |
|  | 0 | 0.76 | 0.5 | 0.48 |  |  | 0.51 |
|  | 0 | 0.91 | 1 | 0.51 |  |  | 0.51 |
|  | 0 | 0.31 | 2 | 0.67 |  |  | 0.51 |
|  | 0.5 | 0.81 | 0.5 | 0.67 |  |  | 0.51 |
|  | 0.5 | 0.86 | 1 | 0.84 |  |  | 0.51 |
|  | 0.5 | 0.26 | 2 | 0.94 |  |  | 0.52 |
|  | 1 | 0.54 | 1 | 0.71 |  |  | 0.52 |
| (Squared) | 1 | 0.20 | 2 | 0.54 |  |  | 0.53 |
|  | 2 | 0.04 | 2 | 0.42 |  |  | 0.54 |
| Cubic |  |  |  |  |  |  |  |
|  | 1 | 0.10 | 2 | 0.06 | 3 | 0.05 | 0.57 |

Table S3 Goodness of fit for meta-regression models
