# Supplementary figures and images for: A cut-off of daily sedentary time and all-cause mortality in adults: a meta-regression analysis involving more than 1 million participants
Source: BMC Med. 2018 May 25;16:74. doi: 10.1186/s12916-018-1062-2 (PMC5998593; doi:10.1186/s12916-018-1062-2)

### Funnel Plot of Standard Error by Log rate ratio

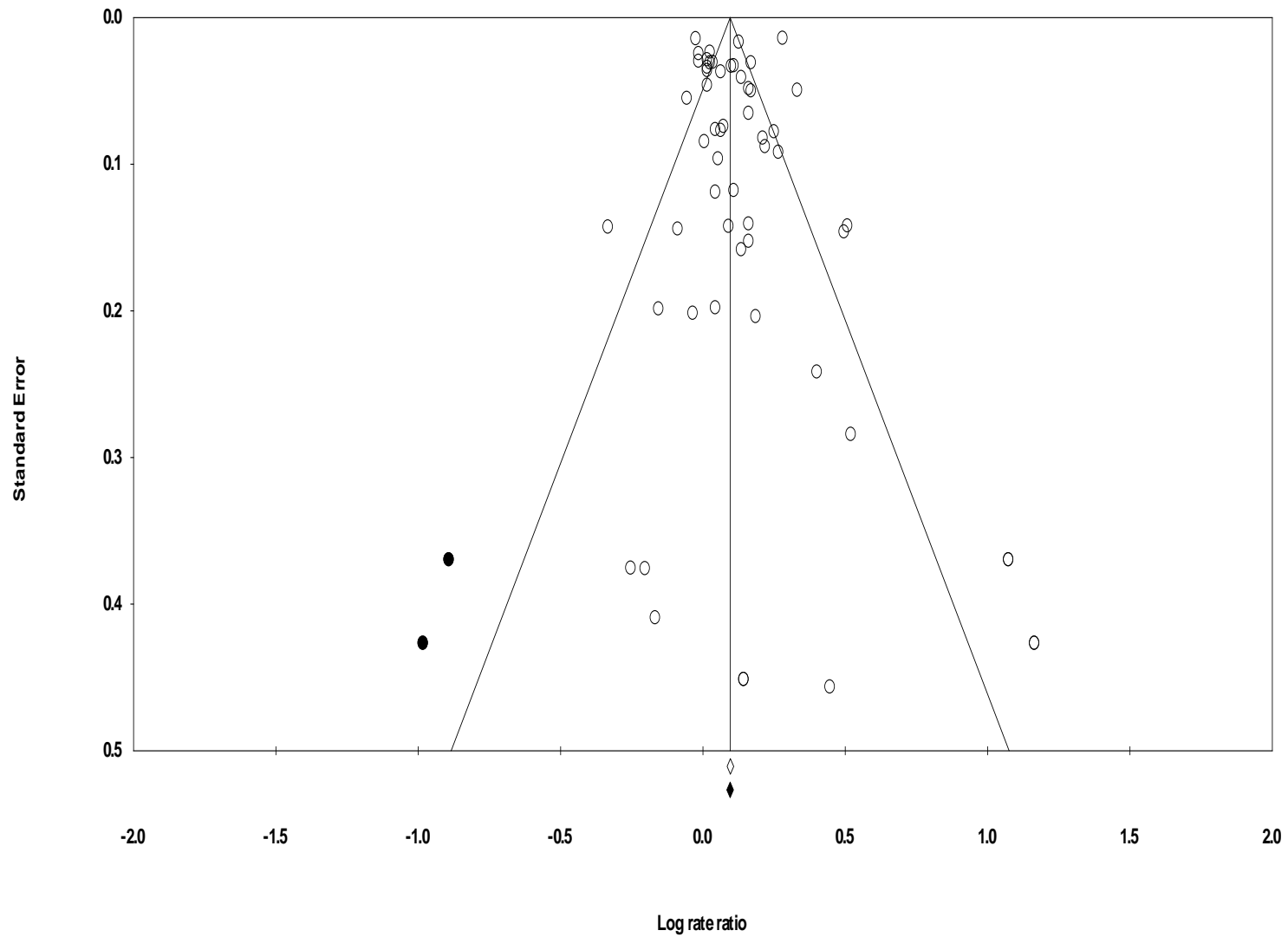

Supplement: Supplementary file 4 — Figure S1. Funnel plot of standard error by log rate ratio. (PDF 7 kb) [file 12916_2018_1062_MOESM4_ESM.pdf]
